# Structural and genomic insights into erythromycin and clindamycin resistance of group B *Streptococcus* isolates in rural West Virginia, United States

**DOI:** 10.3389/fmicb.2025.1686688

**Published:** 2025-11-28

**Authors:** Annabelle P. Smith, Lillie M. Powell, Amaira M. Palmer, Soo Jeon Choi, Rita Berisio, Ryan Demkowicz, Slawomir Lukomski

**Affiliations:** 1 Department of Microbiology, Immunology, and Cell Biology, West Virginia University School of Medicine, Morgantown, WV, United States; 2 Institute of Biostructure and Bioimaging, National Research Council, CNR, Naples, Italy; 3 Department of Pathology, West Virginia University School of Medicine, Morgantown, WV, United States; 4 West Virginia Clinical and Translational Science Institute, Morgantown, WV, United States

**Keywords:** GBS resistance, *Streptococcus agalactiae*, ErmA/B structure, macrolide resistance, lincosamide resistance

## Abstract

**Introduction:**

Group B *Streptococcus* (GBS) is a leading etiological agent of neonatal sepsis and meningitis, as well as invasive infections that predominately affect adults over 65 and people with comorbidities. The Centers for Disease Control and Prevention’s (CDC) nationwide surveillance has identified rising clindamycin and erythromycin resistance as a concerning level threat for invasive GBS infections. West Virginia (WV) is a rural state outside the CDC catchment area, therefore, we investigated 65 WV-GBS isolates identified in clinical specimens from various sources for serotype distribution, as well as the spectrum and genetic markers of erythromycin and clindamycin resistance.

**Results:**

GBS isolates of serotypes II (27.7%), Ib (23.1%), V (18.5%), Ia (13.8%), III (10.8%), and IV (6.2%), were identified. The *mef*(A) gene encoding macrolide resistance (M phenotype) was detected in 15.4% isolates. The *erm*(A) and *erm*(B) genes that are responsible for the combined erythromycin and clindamycin resistance, which characterizes an MLS_B_ [macrolide, lincosamide, and streptogramin B] resistance phenotype, were detected in 67.7% of WV-GBS isolates; also, 81.4% of those displayed constitutive (cMLS_B_) and 18.6% inducible (iMLS_B_) resistance to clindamycin by erythromycin using the D-test. A cluster of mutations within the regulatory region of *erm*(A) were identified in association with a cMLS_B_ sub-phenotype, whereas most of the *erm*(B) promoters sequenced from isolates with a cMLS_B_ background lacked analogous sequence polymorphisms. Further, higher erythromycin MIC values were associated with the *erm*(B) determinant compared with *erm*(A), while structural models of the GBS-ErmA and GBS-ErmB enzymes show conservation in both SAM- and rRNA-binding sites.

**Significance:**

Our data demonstrate that the 80.0% rate of erythromycin and 70.8% of clindamycin resistance in WV is higher than the national average of 61.7 and 52.5%, respectively, while being widespread across a variety of clinical specimens (urine, throat, respiratory tract, blood, foot ulcers, perisplenic fluid, various wounds, and the rectovaginal area). Providers should be aware of the current threat of antibiotic resistance, especially in “primary care deserts” existing in rural areas such as WV.

## Introduction

*Streptococcus agalactiae*, or group B *Streptococcus* (GBS) is a *β*-hemolytic bacterium, which is part of the normal vaginal and lower gastrointestinal flora in 5–30% of adults (Armistead et al., 2019; Van Kassel et al., 2021). The risk of GBS to neonates is severe, as infection can result in lethal sepsis and meningitis. Globally, GBS infections are responsible for approximately 100,000 infant deaths each year (Raabe and Shane, 2019; Stephens et al., 2023). Pregnant women with a positive GBS screen test are administered intrapartum antibiotic prophylaxis (IAP) during labor to prevent development of infection in the newborn. According to the American College of Obstetrics and Gynecology the standard of care is to use penicillin as a first line IAP treatment. However, in cases where the mother has a high-risk penicillin allergy, in which anaphylaxis is a concern, clindamycin is recommended; while erythromycin is avoided due to high rates of resistance (Back et al., 2012; Dhudasia et al., 2021). In addition to neonatal disease, the prevalence of invasive infections in non-pregnant adults has increased, contributing to the incidence of bacteremia, sepsis, and pneumonia, for which approximately 8.0% of cases result in death (Francois Watkins et al., 2019). The age-related decline in immune function for adults 65 and older places this population at higher risk for GBS infections (Francois Watkins et al., 2019). In addition, comorbidities like diabetes are associated with a higher risk for invasive disease (Keogh and Doran, 2023; Monica, 2001). An aging population, growing antibiotic resistance, and high-risk comorbidities have contributed to the rise in these infections (Keogh and Doran, 2023; Monica, 2001).

Since 2006, CDC surveillance determined that erythromycin and clindamycin resistance has steadily increased to a concerning threat level, with estimates that over 50% of isolates were resistant to either antibiotic or both by 2019 (Prevention CfDCa, 2019). In *β*-hemolytic streptococci the *erm*(A) and *erm*(B) methyltransferase genes – encoding enzymes that methylate a shared ribosomal drug binding site - are commonly associated with a MLS_B_ [macrolide (erythromycin), lincosamide (clindamycin), and streptogramin B] resistance phenotype. Additionally, the MLS_B_ phenotype can be further differentiated by a D-test, which is performed in the clinical laboratory to determine whether an isolate has an inducible (iMLS_B_) or constitutive (cMLS_B_) sub-phenotype (Jorgensen et al., 2011). Whereas the *mef* gene, encoding an efflux pump component, is responsible for resistance to erythromycin alone, which is referred to as the M phenotype (Berbel et al., 2022).

The expression of ErmA and ErmB methyltransferase enzymes is regulated by the promoter regions of their corresponding genes and encode two leader peptides L1/L2 and hairpin structures formed by inverted repeats (IR) that collectively regulate ErmA/B production (Ramu et al., 2009). Additionally, a third hairpin is formed by the IR5-IR6 repeats in the *erm*(A) promoter (Fines et al., 2001; Ramu et al., 2011). In the absence of an inducing MLS_B_ antibiotic, enzyme production is inhibited by the formation of two hairpins, through IR1-IR2 and IR3-IR4 pairing in the *erm*(A/B) promoters; the formation of these mRNA structures prevents translation by sequestering the corresponding *erm*-gene Shine-Dalgarno sequences within posterior hairpins (Fines et al., 2001; Wang et al., 2021).

West Virginia (WV) is the 3rd most rural state in the United States of America, wherein the risk for antibiotic misuse is increased (State Office of Rural Health, 2018; Yau et al., 2021). While WV consistently ranks in the top two states for the highest overall number of antibiotic prescriptions (Centers for Disease Control and Prevention, 2022; Services USDoHaH, 2022; State Office of Rural Health, 2018) - to the best of our knowledge, GBS antibiotic resistance in WV has not been explored. Therefore, we analyzed WV-GBS isolates from a variety of clinical specimens by identifying the serotype distribution, genetic markers of erythromycin and clindamycin resistance, and resistance mechanisms. We sequenced the *erm*(A) and *erm*(B) promoters for polymorphisms to determine the implications on mRNA hairpin formation and MLS_B_ sub-phenotype, as well as compared the ErmA and ErmB structural models to explore the observed MIC differences in resistant GBS isolates.

## Results

### Isolate collection and serotyping

Sixty-five WV-GBS isolates were recovered from clinical specimens of different individuals without pre-selection of medical condition or recovery site. Most (n = 21, 32.3%) isolates originated from urine samples. Eleven (16.9%) isolates were identified from routine screening of the rectovaginal region in pregnant women done between 35- and 37-weeks’ gestation. Fourteen (21.5%) isolates were found in throat specimens, whereas two additional isolates were recovered from a tracheal aspirate and bronchoalveolar lavage. Additional isolation sites included blood (*n* = 7), foot ulcers (*n* = 2), perisplenic fluid (*n* = 2), as well as single isolates from the toe, a leg wound, a neck wound, a foot wound, a groin abscess, and the sternocleidomastoid muscle (Supplementary Table S1). Isolates were further classified into invasive, commensal, and “other” based on the range of clinical presentations (Supplementary Table S1). Isolates causing unambiguously invasive disease including bacteremia (7 isolates), splenic abscess (2 isolates), lower extremity ulcers (2 isolates), and deep soft tissue infections (3 isolates) were included in the invasive category. Isolates that were clearly detected in a non-infectious presentation were placed into the colonization category including isolates of the nasopharynx (14 isolates), and anogenital tract (12 isolates). For some isolates, the clinical implications were unclear based on available data. Two GBS isolates from respiratory specimens (tracheal aspirate and bronchoalveolar lavage from Supplementary Table S1) were the predominant organism recovered, but it was unclear if this was commensal flora or the cause of disease. Similarly, three soft tissue wounds included GBS among other organisms in a mixed infection. It is difficult to be definitive about the clinical impact of GBS in these cases. Lasty, urine cultures are continually a point of debate in what classifies as a true urinary infection. Our isolates range from low level bacteriuria without symptoms to frank dysuria with high levels of bacteria present. Information on individual isolates can be found in Supplementary Table S1.

**Figure 1:**
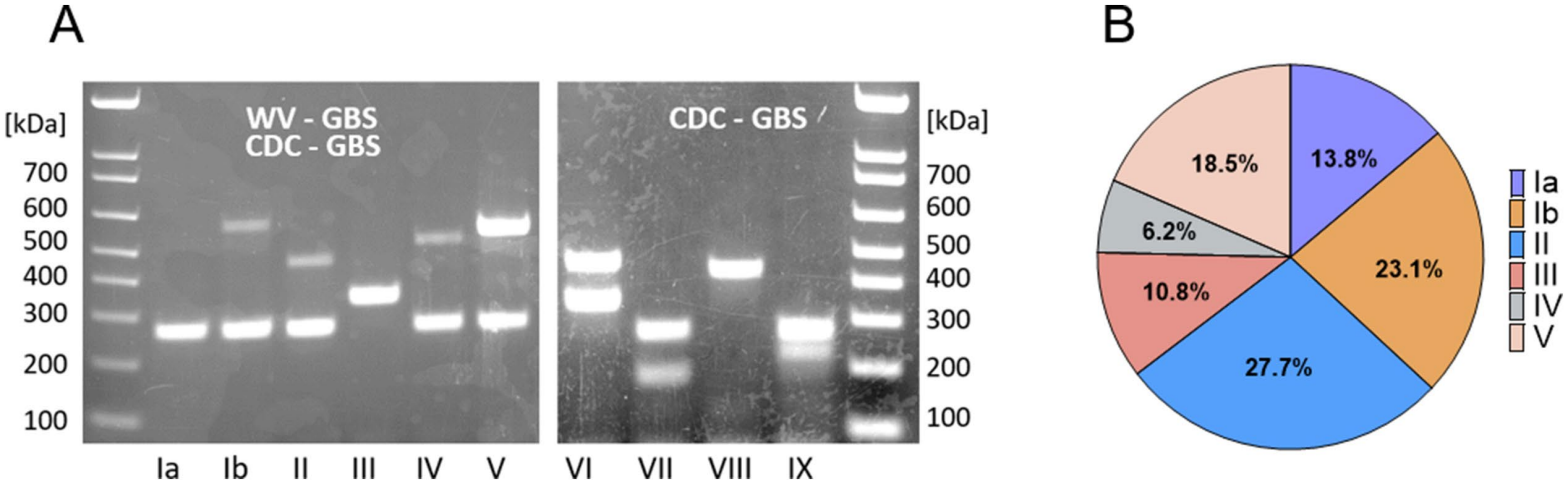
Serotype distribution of WV-GBS isolates. **(A)** Serotype patterns Ia-V of WV-GBS isolates, and VI-IX of CDC-GBS controls as identified by 1.5% agarose gel electrophoresis. **(B)** Graphical depiction of serotype distribution of WV-GBS isolates.

The GBS serotype is assigned based on the unique profile of encoded capsular polysaccharide genes (Imperi et al., 2010). We developed a two-component multiplex assay to foster rapid serotype recognition (Figure 1A). We identified isolates of serotypes Ia through V in the WV-GBS collection, as compared to CDC-GBS controls (Figures 1A,B). The most predominate serotype was II consisting of 18 (27.7%) isolates, followed by: Ib (*n* = 15, 23.1%), V (12, 18.5%), Ia (9, 13.8%), III (7, 10.8%), and IV (4, 6.2%) (Table 1; Figure 1B).

**Table 1:** Characteristics of WV-GBS collection^a^.

	Serotypes						Resistance phenotype				
	Ia	Ib	II	III	IV	V	cMLS_B_	iMLS_B_	M*	SS^+^	Total
Commensal	4	9	7	4	0	2	14	4	2	3	23
Invasive	3	1	3	1	1	5	9	1	2	2	14
Other	2	5	8	2	3	5	12	3	1	8	24
Total	9	15	18	7	4	12	35	8	5	13	61

^a^Information on individual isolates is shown in Supplementary Table S1. * Three isolates (Nos.: 3, 4, and 45 in Supplementary Table S1) are not included due to resistance to clindamycin according to AST results. + One isolate (No 14 in Supplementary Table S1) is not shown due to harboring *erm*(A) but being phenotypically susceptible to clindamycin while resistant to erythromycin.

### Spectrum of erythromycin and clindamycin resistance and genetic determinants

**Figure 2:**
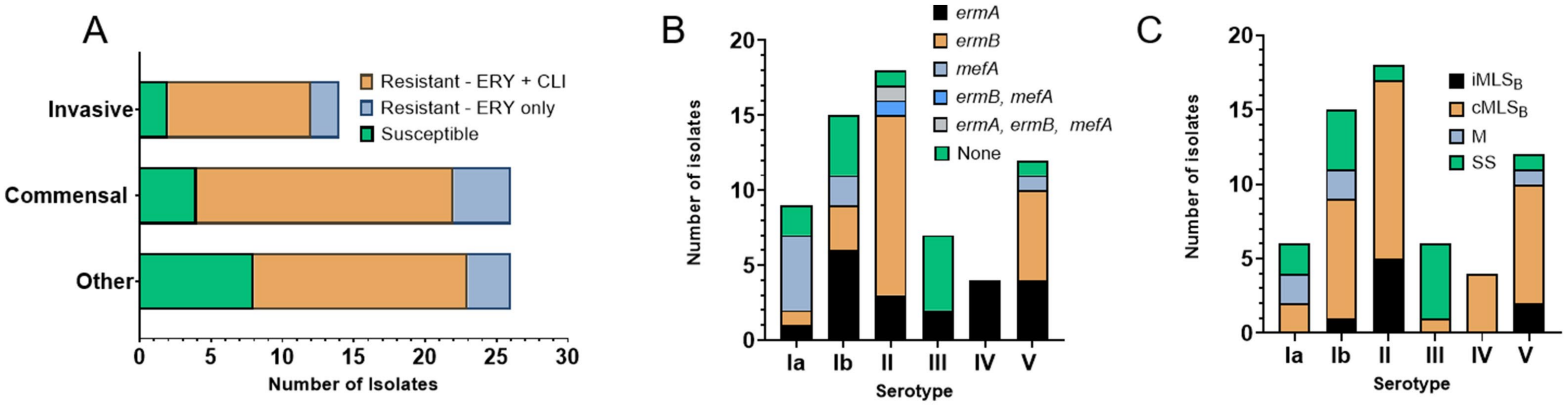
Erythromycin and clindamycin resistance in WV-GBS isolates. **(A)** Graphical depiction of isolates susceptible or resistant to erythromycin and/or clindamycin according to source subcategory, as defined in Supplementary Table S1. **(B)** Graphical depiction of antimicrobial resistance genes by serotype identified by PCR. **(C)** Graphical depiction of resistance sub-phenotypes by serotype, as determined by AST and D-test results. Isolates displaying a non-traditional phenotype were not included (isolates 3, 4, and 45 only harbor *mef*(A) but are phenotypically resistant to clindamycin and isolate 14 harbors *erm*(A) but is phenotypically resistant only to erythromycin. Information on individual isolates can be found in Supplementary Table S1).

The majority of isolates were phenotypically resistant to erythromycin (*n* = 52, 80.0%) and clindamycin (*n* = 46, 70.8%). The percent of isolates, resistant to one or both antibiotics was proportionally the same in the invasive and commensal groups (84.7%) and lower among others (69.2%), which largely contain urine isolates (Supplementary Table S1; Figure 2A). Most serotypes harbored high percentage of resistance genes: serotype Ia (*n* = 7, 77.8%), Ib (*n* = 11, 73.3%), II (*n* = 17, 94.4%), IV (*n* = 4, 100%), and V (*n* = 11, 91.7%), except for III (*n* = 2, 28.6%) (Figure 2B); we acknowledge the overall small number of isolates as a limitation (see discussion section). Most resistant isolates exhibited an MLS_B_ phenotype (*n* = 43, 82.7%), with the *erm*(A) gene harbored in 48.8% (*n* = 21) and the *erm*(B) gene in 55.8% (*n* = 24) (Supplementary Table S1; Figure 2B); one isolate harbored both the *erm*(B) and *mef*(A) genes, while another harbored the *erm*(B), *erm*(A), and *mef*(A) determinants. The *mef*(A) gene alone was detected in 8 (12.3%) isolates (Supplementary Table S1; Figure 2B).

MLS_B_ sub-phenotype classification refers to whether clindamycin resistance is inducible (iMLS_B_) by the presence of an erythromycin disk or if resistance is constitutive (cMLS_B_), according to changes in growth inhibition zones determined in the clinical laboratory by a D-test (Clinical and Laboratory Standards Institute, 2021). In this study, most 81.4% (*n* = 35) of the MLS_B_ resistant isolates exhibited a cMLS_B_ sub-phenotype, corresponding to 87.5% of isolates carrying *erm*(B) and 71.4% isolates with *erm*(A) (Supplementary Table S1; Figure 2C).

Resistance level was further tested in the research laboratory on solid medium with 0–32 μg/mL of erythromycin. Isolates harboring only the *mef*(A) determinant (*n* = 8) had MIC of ≤2 μg/mL Interestingly, 18 isolates harboring *erm*(A) had an MIC of ≤8 μg/mL, and only two had an MIC of >32 μg/mL. Whereas all isolates harboring *erm*(B) (*n* = 24) displayed high-grade resistance with an MIC of >32 μg/mL.

### ErmA and ErmB methyltransferase modeling

**Figure 3:**
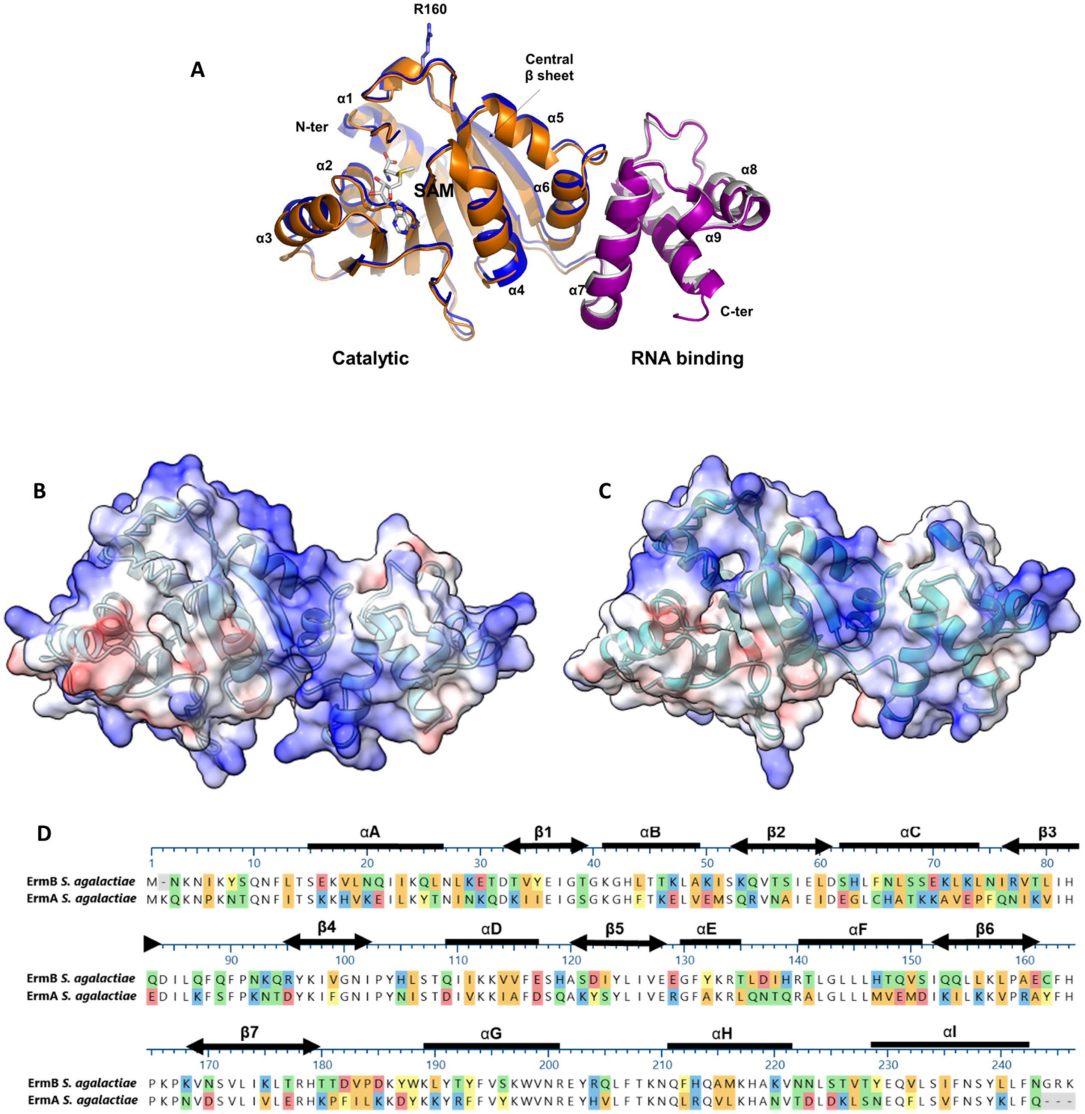
Structural features of the ErmA and ErmB GBS methyltransferases. **(A)** Superposition of ErmA and ErmB structural models. In the cartoon representations, N- (residues 8–181) and C-terminal (residues 182–243) domains of ErmA are drawn in blue and gray, respectively. Homologous domains of ErmB are drawn in orange and prune, respectively. The residue R106 and a SAM molecule are drawn in stick representation. A high level of confidence is predicted for both structures, where the pLDDT scores are >90 for almost all residues of ErmA (residues 11–240) and ErmB (residues 19–158, 168–245). In both cases, the structure of the N-terminal 8 residues is poorly reliable (pLDDT<70) (Bussiere et al., 1998). **(B,C)** Electrostatic potential of the ErmA and ErmB protein models, respectively. The positively charged (blue) concave cleft between the N- and C-terminal domains represents the residues which were predicted to bind the rRNA substrate (Bussiere et al., 1998; Maravic et al., 2003; Sharkey et al., 2022). **(D)** MAFFT alignment of the ErmA and ErmB protein sequences. The secondary structure elements are shown as thick lines for *α*-helices and double-headed arrows for *β*-strands. Colored sections indicate non-conserved residues. The chemistry corresponding to the amino acid classification is shown for each residue as follows: basic (blue), acidic (red), polar uncharged (green), aromatic (yellow), and aliphatic (orange).

To explain disparity between resistance levels associated with either *erm*(A) or *erm*(B) determinants, we used structural modeling to assess whether the GBS-ErmA and GBS-ErmB proteins varied at the moieties crucial for enzymatic activity. Both models display a bi-lobed structure containing a catalytic N-terminal domain and a C-terminal rRNA binding domain (Figure 3A). The N-terminal S-adenosylmethionine (SAM) binding domain adopts an *α*/*β*fold, with a central β sheet surrounded by six α helices, similar to other methyltransferases (Bussiere et al., 1998; Maravic et al., 2003; Sharkey et al., 2022; Yu et al., 1997). The rRNA binding domain is completely α-helical, consisting of three α-helices (αG-αI). ErmA and ErmB present a strongly positive electrostatic surface potential, consistent with the catalytic role of these enzymes (Figures 3B,C). MAFFT protein alignment of both GBS enzymes identified 48% sequence identity with several missense amino acid changes (e.g., Q21E or K71A) that did not alter structure as predicted by modeling. Importantly, the amino acids involved in SAM and rRNA binding were fully conserved (Figure 3D), suggesting that GBS-ErmA and GBS-ErmB function does not differ.

### Regulation of *erm* gene expression

**Figure 4:**
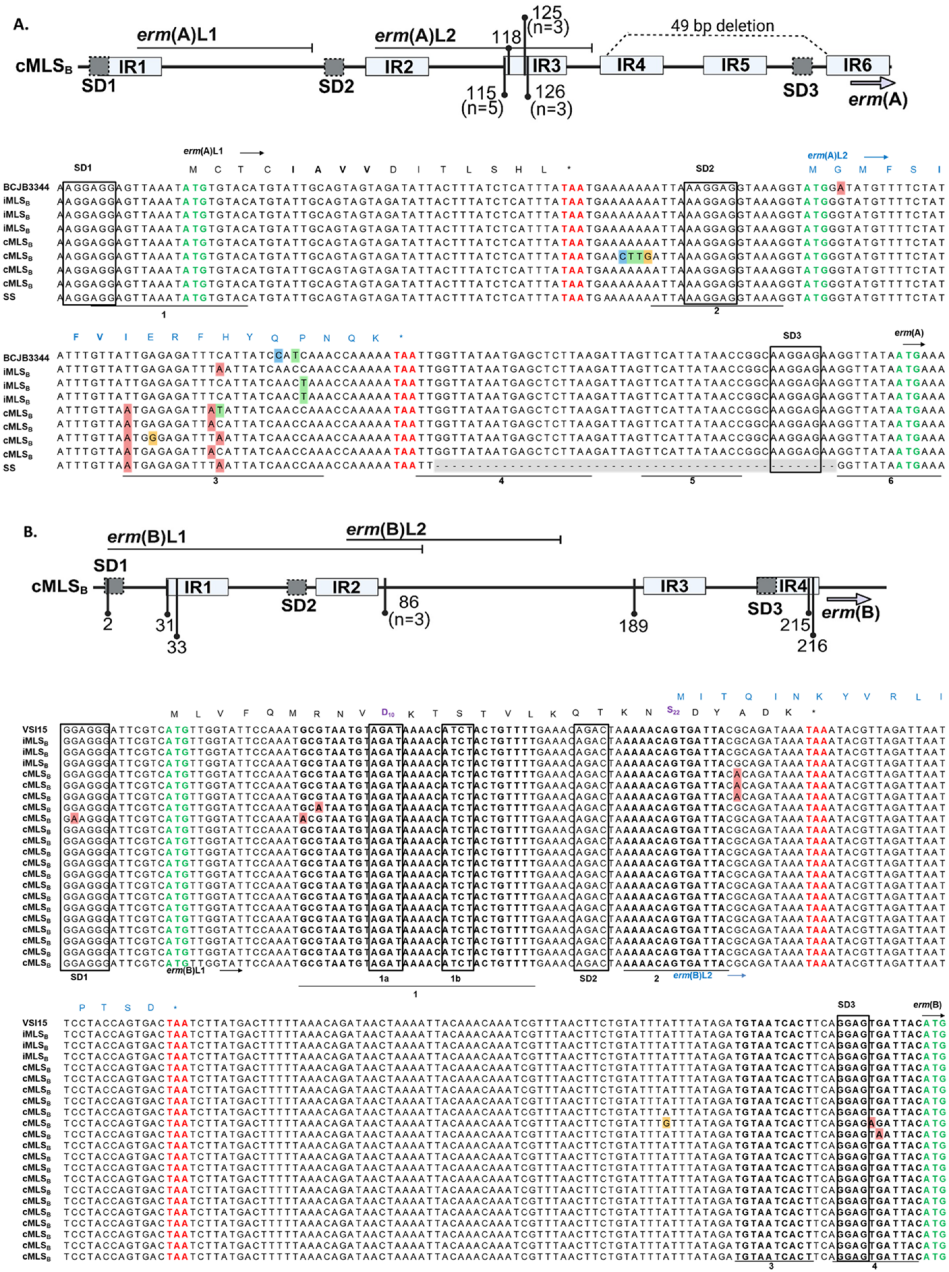
*erm*(A) and *erm*(B) promoter sequence from WV-GBS isolates. Schematic representation of sequence polymorphisms in the *erm*(A) (**A**, top panel) and *erm*(B) (**B**, top panel) regulatory regions (generated with BioRender.com). The *erm* leader peptides [*erm*(A) L1/L2 and *erm*(B) L1/L2] are shown by horizontal lines. Inverted repeats IR1-6 are shown as light gray boxes. The identified point mutations are indicated by vertical lines and numbered according to the distance from the first nucleotide of the Shine Dalgarno (SD1) sequence. Unless otherwise noted in the figure schematics, specific mutations are present in a singular isolate. A 49-bp deletion in the *erm*(A) promoter of a single isolate is shown as a dotted line (**A**, top panel). **(A)** MAFFT multiple sequence alignment of the *erm*(A) promoter from isolates 36, 41, 27, 19, 26, 43, 13 and 14 from top to bottom, for further isolate information see Supplementary Table S1. Sequences shown are labeled according to the AST classification of an isolate as susceptible (SS, *n* = 1) or the D-test classification as either iMLS_B_ (*n* = 3) or cMLS_B_ (*n* = 4). Sequences were compared to the published GBS BCJB3344 genome (Accession: CP101993.1). The ErmAL1 peptide sequence is shown in black text, while the ErmAL2 sequence is annotated by blue text. **(B)** MAFFT multiple sequence alignment of the *erm*(B) promoter. Listed in order of appearance, isolates 38, 33, 11, 31, 22, 50, 58, 24, 44, 9, 26, 45, 56, 40, 35, 1, 39, 58, 66, 60, and 12. Isolate 26 is shown in both alignments as this isolate had acquired *erm*(A) and *erm*(B). Sequences are labeled according to the D-test classification of an isolate as iMLS_B_ (*n* = 3) or cMLS_B_ (*n* = 18), as compared to the published GBS VSI15 genome (Accession: CP118079.1). The ErmBL1 peptide sequence is shown in black text, whereas the ErmBL2 peptide sequence is shown in blue text. The leader peptide sequences motifs responsible for ribosome stalling in *erm*(A) L1 (IAVV) and *erm*(B) L1 (MRNVD_10_ and S_22_) are shown in bold. All Shine Dalgarno sequences are boxed, with the start codon annotated by bold green font and the stop codons of the leader peptides in bold red font.

Sequence polymorphisms in the regulatory region of various *erm* genes have been associated with a cMLS_B_ resistance phenotype (Dzyubak and Yap, 2016; Malhotra-Kumar et al., 2009; Powell et al., 2024; Rosato et al., 1999; Schmitz et al., 2001; Wang et al., 2021; Werckenthin et al., 1999). Previously reported leader peptide and inverted repeat elements were identified in the *erm*(A) and *erm*(B) promoters sequenced from our WV-GBS isolates (Fines et al., 2001; Wang et al., 2021). Here, sequencing identified a cluster of point mutations within the GBS-*erm*(A) IR3 region of isolates with a cMLS_B_ phenotype - affecting the stability of the IR3-IR4 hairpin –promoting IR4-IR5 pairing to render the *erm*(A) SD3 constantly accessible, thus, allowing constant ErmA production (Figure 4A). An additional string of 4 mutations (68–71 A ➔ C, T, T, and G) was identified outside of IR elements in a single cMLS_B_ isolate. Each iMLS_B_ sequence contained single nucleotide polymorphisms in the IR3 region [126 C → A (n=1), 136 C → T (n=2)], compared to the deposited sequence (Accession: CP101993.1). In isolates harboring *erm*(B), the majority with a cMLS_B_ phenotype (*n* = 18, 85.7%) showed no mutations compared to the deposited reference (Accession: CP118079.1; Figure 4B). The remaining 7 cMLS_B_ isolates contained single nucleotide polymorphisms with an unclear impact on IR pairing - both examples suggest unknown alternative mechanisms for the cMLS_B_ phenotype in GBS isolates harboring the *erm*(B) gene.

## Discussion

CDC nationwide surveillance has identified rising GBS resistance to clindamycin and erythromycin as a concerning level threat, although clinico-epidemiological data was collected for invasive GBS infections primarily among urban populations (Prevention CfDCa, 2019). There are unique challenges in rural settings, especially regarding invasive infections, such as distance to care, access to specialists, more difficulty in tracking and reporting, to name a few (Yau et al., 2021). West Virginia (WV) is a rural state outside the CDC catchment area, therefore, we investigated WV-GBS isolates identified in clinical specimens from various sources for serotype distribution, as well as the spectrum, genetic markers, and mechanisms of antibiotic resistance. To our knowledge WV-GBS resistance to erythromycin and clindamycin has not been explored.

Most of our isolates displayed resistance to erythromycin (80.0%) and clindamycin (70.8%), exceeding the national rates of 61.7 and 52.5%, respectively (Centers for Disease Control and Prevention, 2023), which may be explained in part by the difference in communities surveyed as WV has a predominantly rural population where antibiotics are prescribed at the highest rate in the country (Centers for Disease Control and Prevention, 2022). One study from 2019, which looked at data from WV Medicaid patients under 20 to examine antimicrobial stewardship, reported that 201,520 pediatric patients received 227,440 antibiotics that year (Kilgore et al., 2020). Multiple factors contribute to high rates of antibiotic prescription including: less access to specialists, concern of missing an infection, and expectation from patients that they should receive a prescription if they are being seen for sore throat, cough, etc. (Yau et al., 2021). Despite macrolides and clindamycin not being used routinely in treatment of GBS, these antibiotics are used frequently in other cases (Centers for Disease Control and Prevention, 2023). For example, macrolides are often prescribed for upper respiratory infection (e.g., azithromycin), so while GBS is not targeted specifically with these prescriptions, GBS (in the GI tract or vaginal flora) is still exposed to the antibiotic, thus, increasing the potential for resistance to develop. Further, clindamycin has frequently been used for streptococcal or staphylococcal skin/soft tissue infection in patients with *β*-lactam allergies, as well as for dental infections (Berbel et al., 2022). Hence, both macrolides and clindamycin are historically two of the top antibiotics prescribed for mild acute infections leading to overexposure (Del Rosso et al., 2024; Dinos, 2017). The highest number of antibiotic prescriptions in WV are prescribed predominately in outpatient settings both in primary care offices and acute care settings (urgent care/ERs) (Kilgore et al., 2020). The evidence of increased antibiotic resistance in WV for a variety of infectious sources can be seen in the state’s 2024 report on multi-drug-resistant bacteria, which identified an average of 20 cases per 100,000 people, substantially higher than the national average of 3 cases per 100,000 between 2019 and 2023 (Rankin et al., 2025; West Virginia Department of Health, 2024). A 2025 meta-analysis of GBS in 57 countries reports global rates of erythromycin resistance to be 35.0% and clindamycin to be 29.3% with highest rates of resistance for erythromycin in an “undefined area” category (88.9%) and for clindamycin in Nigeria (76.2%) (Hsu et al., 2025).

The lowest levels of erythromycin resistance were displayed by isolates harboring only the *mef*(A) gene ≤2 μg/mL, which is expected with the M phenotype (Berbel et al., 2022; Chaudhary and Piya, 2021). Isolates harboring either the *erm*(A) or *erm*(B) genes had a similar number of occurrences but presented with different MIC-levels of resistance. Isolates containing *erm*(B) consistently displayed a higher level of erythromycin resistance >32 μg/mL, while isolates harboring *erm*(A) displayed lower resistance levels of ≤8 μg/mL. This observation could not be explained by our structural studies of the ErmA and ErmB enzymes, as conservation was exhibited for the SAM co-factor and rRNA binding sites at both the sequence and structural level. The lack of variability between the crucial active sites in ErmA and ErmB indicates that there would be minimal differences in enzyme capability from a structural perspective (Maravic et al., 2003; Sharkey et al., 2022). In the future, additional studies focused on gene expression or ribosome methylation could provide a better understanding of the mechanistic differences that affect MIC.

Our collection consisted of considerably more isolates displaying a cMLS_B_ sub-phenotype of resistance than iMLS_B_. Despite high similarity between the core genomes of *Streptococcus agalactiae* and *Streptococcus pyogenes* (Lee and Andam, 2022; Tettelin et al., 2002), reports support our findings of a cMLS_B_ phenotype being more common in GBS (Chaudhary and Piya, 2021), whereas for GAS the cMLS_B_ phenotype is rare (Leclercq, 2002; Sauermann, 2003). Here, we identified that 81.4% of the GBS isolates with MLS_B_ resistance had a cMLS_B_ sub-phenotype, which is substantially higher than the 13.0% previously observed in our WV collection of *Streptococcus pyogenes* isolates (Powell et al., 2024). However, constitutive expression of Erm enzymes is broadly thought to result in a negative fitness cost due to constant ribosome methylation, where studies have demonstrated this experimentally in an *Escherichia coli* or *Staphylococcus aureus* background (Dzyubak and Yap, 2016; Gupta et al., 2013; Vazquez-Laslop and Mankin, 2018). Altogether, leaving the questions of why a cMLS_B_ phenotype is increased in GBS and what effect this has on bacterial physiology yet to be investigated.

Further, naturally acquired polymorphisms in the regulatory region sequences of the *erm*(A), *erm*(B), *erm*(C), and *erm*(T) genes have been associated with a cMLS_B_ resistance phenotype in various organism backgrounds (Dzyubak and Yap, 2016; Malhotra-Kumar et al., 2009; Powell et al., 2024; Rosato et al., 1999; Schmitz et al., 2001; Wang et al., 2021; Werckenthin et al., 1999). This is supported here by our identification of several single point mutations in the *erm*(A) promoter of cMLS_B_ isolates, that disrupt regulatory hairpin formation, thus allowing constant ErmA production. However, here and in our previous collection of invasive WV-GAS isolates (Powell et al., 2025), we identified that the *erm*(B) promoter sequence of isolates with a cMLS_B_ phenotype were not always associated with mutations. A similar finding was reported by Rosato et al., where only a fraction of the studied enterococcal and streptococcal isolates had polymorphisms that would explain the cMLS_B_ phenotype (Rosato et al., 1999). Altogether, this is indicative that regulation of *erm*(B) expression is not solely attributable to leader peptides and mRNA-hairpin structures. Considering that the presence of *erm*(B) was associated with a higher level of erythromycin resistance compared to the MIC of isolates harboring *erm*(A), despite the lack of variability in enzyme active sites, this calls for the investigation of additional regulatory mechanism(s) to better understand differences in MLS_B_ sub-phenotype expression and their implications on MIC.

Our objective was to gain a demonstrative sample of GBS isolates in WV with no preselection criteria employed, therefore allowing isolates from a variety of sources to be analyzed. We acknowledge limitations to our study, including sample size and the combining of multiple isolates into an “other” group. For a portion of isolates in the collection, it was unclear of the clinical implications. For example, in three soft tissue wounds GBS was identified in addition to other organisms, thereby, it was difficult to definitively assign the clinical impact of GBS in these cases. Whereas invasive GBS infections were considered relatively rare until recently (Francois Watkins et al., 2019), these infections are accompanied by certain immunocompromising comorbidities, such as diabetes, age-related immune decline, and immunodeficiency disorders (Ali et al., 2022; Francois Watkins et al., 2019; Nguyen et al., 2021). The most prevalent source of isolates in our collection was urine. GBS is a natural colonizer of the genitourinary tract therefore, it is often present in urine samples in small amounts (Van Kassel et al., 2021); it is common to see patients that are immune compromised or elderly presenting with UTI symptoms due to GBS (Dobrut et al., 2022; High et al., 2005). Urine cultures are a constant point of debate in what classifies as a true urinary infection, prompting us to place all of these isolates into the “other” category. Here, urine isolates were collected from individuals with and without UTI symptoms, resulting in a range of presentations from low level bacteriuria to dysuria with high levels of bacteria present. Fourteen isolates were collected from the throat, suggesting asymptomatic colonization (Roloff et al., 2018), as GBS throat infections have been infrequently identified (Corrado et al., 1981). The two remaining GBS isolates collected from the respiratory tract were identified as commensal flora, but it is unclear if this was responsible for disease.

In summary, we present findings analyzing the root-mechanisms underlying MLS_B_ resistance using a diverse source collection of GBS isolates from the underrepresented rural population of West Virginia. Considering higher MIC associated with *erm*(B) and a frequent cMLS_B_ sub-phenotype, additional investigations are warranted into whether *erm*-mediated resistance and resulting ribosomal methylation may impact GBS physiology. Clinically, our work shows a high occurrence of erythromycin and clindamycin resistance among GBS isolates from various sources in states like WV - that indicate unique challenges in rural settings including distance to care, access to specialists, more difficulty in tracking/reporting, etc. - suggesting a need for close monitoring of antibiotic resistance in non-urban regions, and better implementation of antimicrobial stewardship practices to avert overexposure and selection of emerging (multi)resistant strains.

## Materials and methods

### Isolate collection

Group B *Streptococcus* (GBS) isolates were identified in clinical specimens submitted to the J. W. Ruby Memorial Hospital Clinical Microbiology Laboratory in Morgantown, WV, which serves as the primary reference facility for 24 West Virginia University Medicine (WVU Medicine) system hospitals and clinics located across West Virginia, as well as western Maryland, southwestern Pennsylvania, and eastern Ohio. GBS isolates analyzed here were recovered from all types of specimens that were submitted over a 4-month period from June to September 2024; several additional blood isolates were recovered from the clinical microbiology laboratory freezer bank that were collected between January–March of 2024. Detection of *β*-hemolytic GBS isolates in clinical samples was performed on sheep blood agar, and ultimately identified by mass spectrometry with the VITEK MS microbial identification system equipped with MALDI-TOF technology (Biomerieux Marcy-l’Étoile, France). Patient clinico-epidemiological data was extracted from the Epic database and reviewed with Institutional Review Board approval (protocol no. 2410057753). Control GBS strains representing all Ia-IX serotypes and those harboring the *erm*(A)*/*(B), and *mef* genes were obtained from the CDC *Streptococcus* Laboratory; more information on strains can be found in Supplementary Table S4. Isolates were collected weekly from the clinical laboratory and immediately underwent DNA isolation and conventional PCR analysis.

### Capsular serotyping

To identify GBS capsular serotypes (Ia, Ib, II-IX), we developed a simplified multiplex PCR protocol with primer sets (Supplementary Table S2) derived from the method described (Imperi et al., 2010), using chromosomal DNA isolated with gBAC Mini Genomic DNA Kit designed for Gram-positive bacteria (IBI Scientific, Dubuque, Iowa, United States - IB47291). Two separate conventional PCR multiplex reactions were carried out under the same conditions with one containing the primers for genes *cpsG*, *cpsN*, and *cpsI*; and the second reaction containing *cpsJ* primers. Samples were amplified after denaturation for 5 min at 95 °C, followed by 30 cycles of 95 °C for 60s, 56 °C for 60s, 72 °C for 60s, and a final cycle of 72 °C for 5 min. Equal volumes of the two PCR reactions were combined and resolved on a 1.5% agarose gel visualized with ethidium bromide. Capsular serotype was assigned as compared to size and patterns obtained for control CDC-GBS reference strains.

### Antimicrobial susceptibility testing and analysis of resistance

Antimicrobial susceptibility testing for erythromycin (15 μg) and clindamycin (2 μg), were performed by disk diffusion with BBL™ Sensi-Disk™ (BD Biosciences, Franklin Lakes, New Jersey, United States) on Mueller Hinton Agar (BD Biosciences, Franklin Lakes, New Jersey, United States) according to the Clinical Laboratory Standards Institute (CLSI M02E14); zone diameter breakpoints were used for interpretation (CLSI M100 Ed34). If applicable, a D-test was performed as described (CLSI M100 Ed34). Briefly, any degree of clindamycin zone flattening in proximity to the erythromycin disk was interpreted as a positive result. D-test positive isolates were referred to as inducible (iMLS_B_), whereas isolates with confluent growth around both the erythromycin and clindamycin disks were considered to have a constitutive (cMLS_B_) sub-phenotype. The degree of erythromycin resistance was assessed in the research laboratory on brain heart infusion (BHI) agar (BD Biosciences, Franklin Lakes, New Jersey, United States) containing erythromycin concentrations of 2, 4, 8, 16 and 32 μg/mL. Overnight GBS cultures grown in Todd Hewitt broth (BD Biosciences, Franklin Lakes, New Jersey, United States) were diluted 1:100 and 10-μL aliquots were plated onto BHI agar containing the antibiotic; bacterial growth was assessed after overnight incubation at 37 °C with 5% CO_2_.

The resistance genes *erm*(A), *erm*(B), and *mef*(A) were detected using primers as described (Supplementary Table S3) (Dilrukshi et al., 2023). Samples were amplified after denaturation for 1 min at 95 °C; 35 cycles of 95 °C for 40s, 59 °C for 40s, 72 °C for 60s; and 72 °C for 5 min then visualized with ethidium bromide on a 1% agarose gel along with CDC-GBS controls. The regulatory promoter regions of the *erm*(A) and *erm*(B) genes were PCR-amplified using primers described (Supplementary Table S3) (Seppälä et al., 1998). Samples were amplified after denaturation for 5 min at 95 °C; 30 cycles of 95 °C for 30s, 50 °C for 45s, 72 °C for 30s; and a final cycle of 72 °C for 5 min sequenced and analyzed using Lasergene DNAStar MegAlign17 software; the MAFFT alignment algorithm was used to detect polymorphisms.

### Structural modeling of Erm methyltransferases

The 3D structures of ErmA and ErmB were computed using the AlphaFold3.0 (AF) (Abramson et al., 2024). The reliability of the AF predictions was assessed by the Local Distance Difference Test (LDDT) score (0–100), a per-residue confidence score, with values greater than 90 indicating high confidence, and low confidence below 50. Structures were analyzed and displayed using PyMOL (Seeliger and De Groot, 2010) and ChimeraX (Pettersen et al., 2021).

## Data Availability

The original contributions presented in the study are included in the article/Supplementary material, further inquiries can be directed to the corresponding author/s.
