# Supplementary material for: Structural and genomic insights into erythromycin and clindamycin resistance of group B Streptococcus isolates in rural West Virginia, United States
Source: Front Microbiol. 2025 Nov 28;16:1686688. doi: 10.3389/fmicb.2025.1686688 (PMC12698558; doi:10.3389/fmicb.2025.1686688)
Supplement: Supplementary file 1 [file Table_1.docx]

Supplementary Material

**Table S1:** Collection and characteristics of isolates sorted by source

| Source | Isolate # | Serotype | Genetic elements | Resistance phenotype | Sub-classification |
| --- | --- | --- | --- | --- | --- |
| Bronchoalveolar lavage | 1 | V | *ermB* | cMLS_B_ | Other |
| Tracheal aspirate | 2 | IV | *ermA* | cMLS_B_ | Other |
| Throat | 3 | Ia | *mefA* | *^1^*R | Commensal |
|  | 4 | Ia | *mefA* | *^1^*R | Commensal |
|  | 5 | Ib | *ermA* | cMLS_B_ | Commensal |
|  | 6 | Ib | *ermA* | cMLS_B_ | Commensal |
|  | 7 | Ib | *ermA* | cMLS_B_ | Commensal |
|  | 8 | Ib | *-* | SS | Commensal |
|  | 9 | Ib | *ermB* | cMLS_B_ | Commensal |
|  | 10 | Ib | *ermA* | cMLS_B_ | Commensal |
|  | 11 | II | *ermB* | iMLS_B_ | Commensal |
|  | 12 | II | *ermB* | cMLS_B_ | Commensal |
|  | 13 | II | *ermB* | cMLS_B_ | Commensal |
|  | 14 | III | *ermA* | *^2^*R | Commensal |
|  | 15 | III | *-* | SS | Commensal |
|  | 16 | V | *ermA* | cMLS_B_ | Commensal |
| Blood | 17 | Ia | *ermA* | cMLS_B_ | Invasive |
|  | 18 | Ia | *mefA* | M | Invasive |
|  | 19 | Ib | *ermA* | cMLS_B_ | Invasive |
|  | 20 | II | *ermA* | cMLS_B_ | Invasive |
|  | 21 | IV | *ermA* | cMLS_B_ | Invasive |
|  | 22 | V | *ermB* | cMLS_B_ | Invasive |
|  | 23 | V | *mefA* | M | Invasive |
| Groin abscess | 24 | II | *ermB* | cMLS_B_ | Invasive |
| Foot ulcer | 25 | Ia | *-* | SS | Invasive |
|  | 26 | II | *ermB, ermA, mefA* | cMLS_B_ | Other |
| Right plantar foot wound | 27 | V | *ermA* | iMLS_B_ | Other |
| Leg wound | 28 | V | *ermA* | cMLS_B_ | Invasive |
| Neck wound | 29 | IV | *ermA* | cMLS_B_ | Other |
| Perisplenic fluid | 30 | V | *ermB* | cMLS_B_ | Invasive |
|  | 31 | V | *ermB* | cMLS_B_ | Invasive |
| Sternocleidomastoid | 32 | III | *-* | SS | Invasive |
| Toe | 33 | II | *ermB* | iMLS_B_ | Invasive |
| Pregnancy screen | 34 | Ia | *mefA* | M | Commensal |
|  | 35 | Ia | *ermB* | cMLS_B_ | Commensal |
|  | 36 | Ib | *ermA* | iMLS_B_ | Commensal |
|  | 37 | Ib | *mefA* | M | Commensal |
|  | 38 | II | *ermB* | iMLS_B_ | Commensal |
|  | 39 | II | *ermB* | cMLS_B_ | Commensal |
|  | 40 | II | *ermB* | cMLS_B_ | Commensal |
|  | 41 | II | *ermA* | iMLS_B_ | Commensal |
|  | 42 | III | *-* | SS | Commensal |
|  | 43 | III | *ermA* | cMLS_B_ | Commensal |
|  | 44 | V | *ermB* | cMLS_B_ | Commensal |
|  | 45 | Ib | *ermB* | cMLS_B_ | Commensal |
| Urine | 46 | Ia | *mefA* | *^1^*R | Other |
|  | 47 | Ia | *-* | SS | Other |
|  | 48 | Ib | *-* | SS | Other |
|  | 49 | Ib | *-* | SS | Other |
|  | 50 | Ib | *ermB* | cMLS_B_ | Other |
|  | 51 | Ib | *-* | SS | Other |
|  | 52 | Ib | *mefA* | M | Other |
|  | 53 | II | *-* | SS | Other |
|  | 54 | II | *ermA* | iMLS_B_ | Other |
|  | 55 | II | *ermB, mefA* | cMLS_B_ | Other |
|  | 56 | II | *ermB* | cMLS_B_ | Other |
|  | 57 | II | *ermB* | cMLS_B_ | Other |
|  | 58 | II | *ermB* | cMLS_B_ | Other |
|  | 59 | II | *ermB* | cMLS_B_ | Other |
|  | 60 | III | *-* | SS | Other |
|  | 61 | III | *-* | SS | Other |
|  | 62 | IV | *ermA* | cMLS_B_ | Other |
|  | 63 | V | *-* | SS | Other |
|  | 64 | V | *ermA* | iMLS_B_ | Other |
|  | 65 | V | *ermB* | cMLS_B_ | Other |
| *^1^Phenotypically resistant to clindamycin by AST*  *^2^Detection of erm(A) by PCR, however isolate is not phenotypically resistant to clindamycin, thus no MLS_B_ designation* | | | | | |
