# Supplementary material for: Structural and genomic insights into erythromycin and clindamycin resistance of group B Streptococcus isolates in rural West Virginia, United States
Source: Front Microbiol. 2025 Nov 28;16:1686688. doi: 10.3389/fmicb.2025.1686688 (PMC12698558; doi:10.3389/fmicb.2025.1686688)
Supplement: Supplementary file 2 [file Table_2.docx]

Supplementary Material

**Table S2:** Serotyping primers

| Capsule gene | Primer name | Sequence | Reference |
| --- | --- | --- | --- |
| G | GBS-cpsG-F | 5’-ACATGAACAGCAGTTCAACCGT-3’ | (21) |
|  | GBS-CpsG-R | 5’-ATGCTCTCCAAACTGTTCTTGT-3’ |  |
|  | GBS-CpsG-2-3-6-R | 5’-TCCATCTACATCTTCAATCCAAGC-3’ |  |
| N | GBS-CpsN-5-F | 5’-ATGCAACCAAGTGATTATCATGTA-3’ |  |
|  | GBS-CpsN-5-R | 5’-CTCTTCACTCTTTAGTGTAGGTAT-3’ |  |
| I | GBS-cpsI-Ia-6-7-F | 5’-GAATTGATAACTTTTGTGGATTGCGATGA-3’ |  |
|  | GBS-cpsI-6-R | 5’-CAATTCTGTCGGACTATCCTGATG-3’ |  |
|  | GBS-cpsI-7-R | 5’-TGTCGCTTCCACACTGAGTGTTGA-3’ |  |
|  | GBS-cpsI-7-9-F | 5’-CTGTAATTGGAGGAATGTGGATCG-3’ |  |
|  | GBS-cpsI-9-R | 5’-AATCATCTTCATAATTTATCTCCCATT-3’ |  |
| J | GBS-CpsJ-8-F | 5’-TATTTGGGAGGTAATCAAGAGACA-3’ |  |
|  | GBS-CpsJ-8-R | 5’-GTTTGGAGCATTCAAGATAACTCT-3’ |  |
|  | GBS-cpsJ-2-4-F | 5’-CATTTATTGATTCAGACGATTACATTGA-3’ |  |
|  | GBS-cpsJ-2-R | 5’-CCTCTTTCTCTAAAATATTCCAACC-3’ |  |
|  | GBS-cpsJ-4-R | 5’-CCTCAGGATATTTACGAATTCTGTA-3’ |  |
|  | GBS-cpsJ-Ib-F | 5’-GCAATTCTTAACAGAATATTCAGTTG-3’ |  |
|  | GBS-cpsJ-Ib-R | 5’-GCGTTTCTTTATCACATACTCTTG-3’ |  |
