# Supplementary material for: Structural and genomic insights into erythromycin and clindamycin resistance of group B Streptococcus isolates in rural West Virginia, United States
Source: Front Microbiol. 2025 Nov 28;16:1686688. doi: 10.3389/fmicb.2025.1686688 (PMC12698558; doi:10.3389/fmicb.2025.1686688)
Supplement: Supplementary file 3 [file Table_3.docx]

Supplementary Material

**Table S3:** Primers for analysis of antimicrobial resistance

| Gene(s) | Primer | Sequence | Reference |
| --- | --- | --- | --- |
| *erm*(A) | *erm*(A) forward | 5’-GAAGTTTAGCTTTCCTAA-3’ | (54) |
|  | *erm*(A) reverse | 5’-GCTTCAGCACCTGTCTTAATTGAT-3’ |  |
|  | *erm*(A) reg forward | 5’-GCATAAGGAGGAGTTAAATATG-3’ | (55) |
|  | *erm*(A) reg reverse | 5’-CTTCAGCACCTGTCTTAATTG-3’ |  |
| *erm*(B) | *erm*(B) forward | 5’-GAAAAGGTACTCAACCAAATA-3’ | (54) |
|  | *erm*(B) reverse | 5’-AGTAACGGTACTTAAATTGTTTAC-3’ |  |
|  | *erm*(B) reg forward | 5’-TTTTGTGTATAATAGGAATTGAAG-3’ | This study |
|  | *erm*(B) reg reverse | 5’-CATAAGATTAGTCACTGGTAGG-3’ | This study |
| *mef*(A*)* | *mef*(A) forward | 5’- AGTATCATTAATCACTAGTGC-3’ | (54) |
|  | *mef*(A) reverse | 5’- TTCTTCTGGTACTAAAAGTGG-3’ |  |
|  | *mef*(A) reg forward | 5’-ACAAAGATGTAGGAGGAACCG-3’ | This study |
|  | *mef*(A) reg reverse | 5’-GATACTGCTTGCCCTGCCC-3’ | This study |
