# Supplementary material for: Structural and genomic insights into erythromycin and clindamycin resistance of group B Streptococcus isolates in rural West Virginia, United States
Source: Front Microbiol. 2025 Nov 28;16:1686688. doi: 10.3389/fmicb.2025.1686688 (PMC12698558; doi:10.3389/fmicb.2025.1686688)
Supplement: Supplementary file 4 [file Table_4.docx]

Supplementary Material

**Table S4:** CDC control strains

| Serotype | Resistance gene(s) | CDC strain ID | Isolation year |
| --- | --- | --- | --- |
| Ia | n/a | 20221401 | 2021 |
| Ib | *erm(A)* | 20225028 | 2021 |
| II | *erm(B)* | 20215404 | 2021 |
| III | *mef(A)* | 20216283 | 2021 |
| IV | *erm(A)* | 20225049 | 2021 |
| V | *erm(B)* | 20220284 | 2021 |
| VI | n/a | 20220498 | 2021 |
| VII | n/a | 20154176 | 2015 |
| VIII | n/a | 20215779 | 2021 |
| IX | n/a | 20164657 | 2016 |
